# Electroforming‐Free, Self‐Rectifying Selector‐Only Memory With Diffusive Cu‐Ion Dynamics for Logic‐In‐Memory Computing

**DOI:** 10.1002/advs.77423

**Published:** 2026-08-25

**Authors:** Jae‐Kyeong Kim, Dae‐Seong Woo, Min‐Jong Han, Hong‐Gwang Seo, Gwang‐Ho Park, In‐Taek Woo, Jea‐Gun Park, Jinsub Park

**Affiliations:** ^1^ Department of Nano‐scale Semiconductor Engineering Hanyang University Seoul Republic of Korea; ^2^ SK Hynix Inc. Icheon Republic of Korea; ^3^ Department of Artificial Intelligence Semiconductor Engineering Seoul Republic of Korea; ^4^ Department of Electronic Engineering Hanyang University Seoul Republic of Korea; ^5^ Advanced Semiconductor Materials and Devices Development Center Hanyang University Seoul Republic of Korea

**Keywords:** compute‐express‐link, in‐memory computing, logic‐in‐memory, selector‐only memory, self‐rectifying

## Abstract

A selector‐only memory (SOM), performing the dual functions of a selector and memory in a single device, has emerged as a promising candidate to overcome critical issues such as high aspect ratio and thermal disturbance in 3D X‐point arrays (3DXP) for compute‐express‐link (CXL). Nevertheless, previously reported SOMs based on ovonic threshold switching (OTS) still have limitations in adoption as a new memory solution for 3DXP, owing to the requirement of an electroforming process at high voltage, bidirectional high current flow, and the intrinsic drift phenomenon. Here, we report a novel electroforming‐free self‐rectifying SOM (SR‐SOM) that exhibits a diode‐like unidirectional current flow and high endurance of ∼10^8^ cycles. Previous SOM strategies have relied primarily on chalcogenide‐based OTS selectors to induce threshold voltage modulation. By contrast, our SR‐SOM operates via diffusive Cu‐ion dynamics, where the polarity‐induced threshold‐voltage shift originates from atomic migration under an applied bias, and the ultrafast self‐rupturing of conductive Cu filaments enables drift‐free properties with sub‐20‐ns. Moreover, by implementing a logic‐in‐memory scheme, we demonstrated all 16 Boolean logic functions and a full adder operation, verifying the multifunctional capability of SR‐SOM for both memory and logic operations.

## Graphical abstract

Electroforming‐free, self‐rectifying switching with polarity‐dependent threshold modulation is realized in selector‐only memory through Cu‐ion migration in a bilayer stacked dual functional materials. Ultrafast rupturing of Cu filament enables drift‐free operation with high stability. Furthermore, all 16 Boolean logic functions and a full adder were implemented using the selector‐only memory.

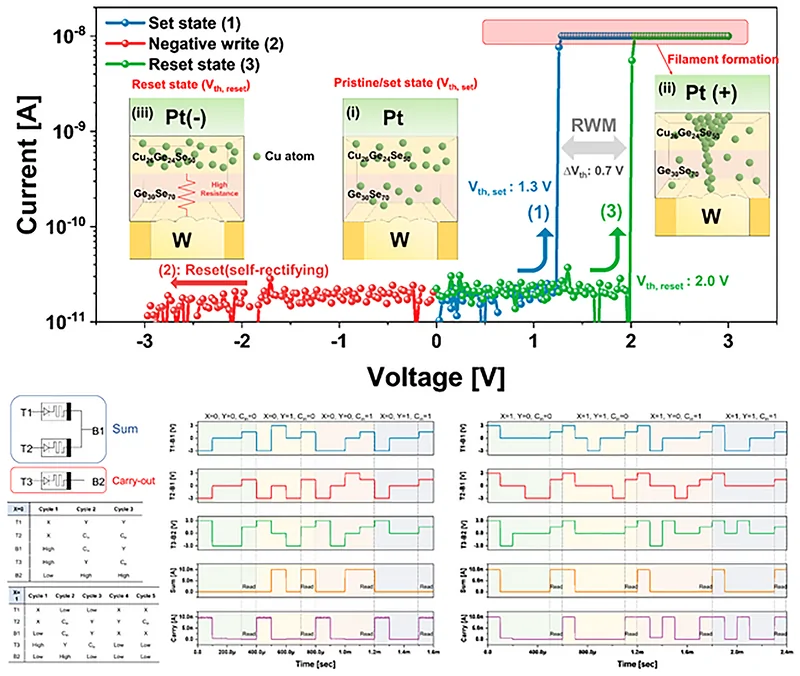

## 1 Introduction

Advances in deep learning technology, enormous amounts of computing resources (e.g., processors and memory), and large‐scale training data have enabled the emergence of large language models (LLMs) such as ChatGPT [1, 2, 3]. These LLMs have been implemented using hyperscale neural networks with billions of parameters trained using large amounts of unlabeled text data via a self‐supervised learning approach, allowing machines to read, write, and communicate with humans through precisely designed artificial intelligence (AI) architectures [4, 5]. To maximize the efficiency of the training and inference of hyperscale neural networks, advanced AI semiconductor technologies capable of processing large amounts of data, sharing memory resources in heterogeneous data‐centric computing, and reducing power consumption during data movement are essential. High‐bandwidth memory (HBM) [6, 7], compute‐express‐link (CXL) [8, 9], and processing‐in‐memory (PIM) technologies [10, 11] have been proposed to realize these requirements. Among these, CXL, a standard interconnect technology, is suitable for the training and inference of hyperscale AI owing to the memory capacity expansion of the central processing unit (CPU) by up to tens of times. Major semiconductor manufacturers have released CXL‐based memory expanders employing dynamic random access memory (DRAM) [12, 13]. However, as the scaling down of DRAM technology has slowed significantly, alternative memory technologies capable of high‐density array operation have emerged as a promising approach for next‐generation CXL systems [14].

As a memory solution for next‐generation CXL, a 3D X‐point array (3DXP) [15, 16] consisting of a phase‐change random‐access memory (PCRAM) [17, 18, 19, 20, 21, 22, 23] and an ovonic threshold switching (OTS) selector [24, 25, 26, 27, 28, 29, 30, 31] has been proposed because of its non‐volatility, fast speed, and high scalability. Nevertheless, vertically stacked high‐density 3DXP still faces limitations owing to the high aspect ratio caused by the serial connection of PCRAM and OTS, and thermal disturbance between adjacent cells by the use of phase change materials [32]. To address these limitations, selector‐only memory (SOM) devices employing polarity‐induced threshold voltage (V_th_) shifts in pure chalcogenide‐based dual‐functional materials (DFM) have been explored, enabling the simultaneous realization of selector and memory functionalities within a single device structure [32, 33, 34, 35]. Previously reported SOMs mainly used pure GeAsSe or doped GeAsSe (e.g., SiGeAsSe or SiTeGeAsSe) chalcogenide materials as DFM to improve the thermal stability and electrical reliability, as summarized in Table 1. Although the SOMs reported to date with nanoscale cell diameter have demonstrated high performance of electrical characteristics and reliability, several problems still need to be improved such as the reproducibility of the chemical composition ratio of DFM, the requirement of an electroforming at high voltage, bi‐directional current flow in both positive and negative write process, and the intrinsic drift phenomenon that V_th_ decreases during rapid subsequent programming due to atomic rearrangements in pure chalcogenide materials [36, 37, 38]. These problems can cause various issues, such as the chemical composition ratio reliability of DFM during the deposition process, including physical vapor deposition (PVD) and atomic layer deposition (ALD); the complexity of the peripheral circuit at the chip level due to high electroforming voltage; power consumption of high‐density cross‐point arrays during the programming process due to bidirectional positive and negative write processes; and retention failures and slow subsequent operations by inducing delays due to V_th_ drift. Thus, in this study, we propose a novel SOM that is electroforming‐ and drift‐free, self‐rectifying (i.e., unidirectional current flow), and eco‐friendly DFM without the use of highly toxic arsenic atoms [39].

**TABLE 1:** The material and electrical performance of the reported OTS‐only SOMs and this work.

	Device type	DFM	Cell diameter [nm]	Forming	V_th_ [V]	RWM [V]	Drift	Endurance	Current flow	Refs.
**Hong** **et al**.	OTS‐only		20	—	—	—	O	> 10^7^	Bidirectional	[32]
**Ravsher** **et al**.	OTS‐only	SiGeAsSe	∼75	O (V_forming_: 7 V)	5∼6	1.3	O	> 10^8^	Bidirectional	[33]
**Lee** **et al**.	OTS‐only	SiTeGeAsSe	50 ∼ 100	O (V_forming_: 4.2 V)	3∼4	1.2	O	> 10^8^	Bidirectional	[34]
**Park** **et al**.	OTS‐only	GeAsSe	∼16	—	—	0.5	—	> 10^8^	Bidirectional	[35]
**This work**	Filamentary	CuGeSe/GeSe	∼30	X	<2	∼0.7	X	> 10^8^	Unidirectional	

Herein, we report a self‐rectifying selector‐only memory (SR‐SOM) that exploits diffusive Cu‐ion dynamics within a bi‐layer dual‐functional material (DFM) stack of Cu_26_Ge_24_Se_50_/Ge_30_Se_70_, engineered for next‐generation CXL memory architectures and extended applicability to logic‐in‐memory computing. The 30‐nm‐diameter SR‐SOM exhibits abrupt threshold switching with polarity‐dependent voltages (V_th, set_ of 1.3 V and V_th, reset_ of 2.0 V) without requiring electroforming, thereby ensuring a robust read‐window margin (RWM) of ∼0.7 V under a low operating‐voltage regime of 3 V. While previously reported threshold‐voltage‐tunable Ag_x_Te_1__−__x_/Al_2_O_3_/TiO_2_ serial hybrid device relies on physically separated memory and selector layers [40], the present SR‐SOM takes a fundamentally different approach by exploiting bias‐history‐dependent Cu redistribution within an integrated Cu‐doped chalcogenide bilayer DFM, thereby enabling electroforming‐free, self‐rectifying, and low‐voltage operation. The inherent self‐rectification originates from the high resistivity of the Ge_30_Se_70_ layer, mitigating electrically induced thermal stress and enabling high write/erase endurance up to ∼10^8^ cycles. The mechanism underlying polarity‐induced V_th_ shifting was elucidated using time‐of‐flight secondary ion mass spectrometry (ToF‐SIMS), which revealed Cu‐ion redistribution within the DFM depending on the history of the applied bias. Furthermore, the abrupt threshold‐switching (TS) behavior was elucidated using double‐swept DC current–voltage (I−V) measurements conducted over a wide temperature range (200–400 K). Unlike previously reported OTS‐only SOMs, our SR‐SOM exhibited intrinsic drift‐free switching within 20 ns, which was attributed to the ultrafast self‐rupturing of transient Cu conductive filaments in the DFM layer. Finally, we demonstrate all 16 Boolean logic operations and a full adder through a sequential logic‐in‐memory scheme utilizing one or more SR‐SOM devices, confirming their viability for hardware‐embedded logic execution. Consequently, these results suggest that SR‐SOM is a compelling candidate for next‐generation CXL‐based memory solutions for AI semiconductor systems, with the potential to significantly improve AI training and inference efficiency by alleviating the overload of processing units.

## 2 Design of the SR‐SOM

Unlike the previously reported SOMs operated by the OTS mechanism summarized in Table 1, the SR‐SOM was designed using a filamentary switching mechanism with conductive Cu filaments consisting of a 30‐nm‐thick Pt top electrode, 10‐nm‐thick Cu‐alloyed GeSe, and 10‐nm‐thick GeSe chalcogenide materials on a 30‐nm‐diameter plug‐type W bottom electrode, as shown in the cross‐sectional transmission electron microscope (x‐TEM) image in Figure 1a. In Figure 1a, we denote the bilayer‐stacked Cu‐alloyed GeSe and pure GeSe chalcogenide materials as a DFM acting as reservoirs for Cu ions and resistive switching layers. In addition, Si_3_N_4_ and SiO_2_ layers were used as stop nitrides through a chemical mechanical planarization (CMP) process and as a passivation layer to electrically separate the devices, respectively. The cropped 3D surface morphology of the 30‐nm‐diameter W bottom electrode was scanned by atomic force microscopy, exhibiting a highly uniform root mean square (R_q_) roughness of 0.588 nm by the CMP process, as shown in Figure 1b. In particular, the formation of conductive Cu filaments within the DFM predominantly occurred in the hillock regions of the 30‐nm‐diameter W bottom electrode, where the locally enhanced electric field concentration was significantly higher than that in the valley regions [41, 42]. The atomic composition ratios of the Cu‐alloyed GeSe layer and pure GeSe layer were precisely controlled, resulting in Cu_26_Ge_24_Se_50_ and Ge_30_Se_70_, respectively, as shown by the auger electron spectroscopy depth profiles in Figure 1c. The Se‐rich Ge_30_Se_70_ chalcogenide material was utilized as a resistive switching layer to achieve a low leakage current owing to its high‐energy bandgap (1.29 eV), which was linearly fitted with the Tauc plots in Figure S1a [43]. The energy bandgap of the Cu_26_Ge_24_Se_50_ reservoir layer was decreased slightly to 0.99 eV by alloying metallic Cu atoms into the pure Ge_30_Se_70_ layer, as shown in Figure S1b. In addition, the energy‐level alignment of the Pt/Cu_26_Ge_24_Se_50_/Ge_30_Se_70_/W stack was further investigated by ultraviolet photoelectron spectroscopy (UPS, XPS‐Theta Probe), and the corresponding UPS‐based band alignment and schematic biased energy band diagrams are shown in Figure S2. In the magnified cross‐sectional x‐TEM image in Figure 1d, the bilayer DFM consisting of two compositionally similar chalcogenide materials (Cu_26_Ge_24_Se_50_ and Ge_30_Se_70_) shows no distinct interface. However, most of the Cu atoms in the Cu_26_Ge_24_Se_50_ reservoir layer diffused into the pure Ge_30_Se_70_ layer in the pristine state because of the high sputtering energy during the Cu and GeSe co‐sputtering processes, as shown in the energy‐dispersive x‐ray spectroscopy (EDS) mapping image in Figure 1e. The Pt and W atoms were uniformly distributed in each layer (i.e., the top and bottom electrodes); in particular, the Se atoms were largely distributed within the Se‐rich DFM. Because the Cu atoms largely diffused into the pure Ge_30_Se_70_ layer in the initial state, the pre‐diffused Cu nuclei within the matrix lowered the activation barrier for filament initiation, thereby eliminating the need for an initial formation process, as shown in the DC I‐V curve in Figure 1f. The pristine‐stated SR‐SOM (inset illustration (i) of Figure 1f) performed abrupt threshold switching via formation of conductive Cu filaments (inset illustration (ii) of Figure 1f) at V_th, set_ of 1.3 V without an electroforming process when the voltage bias was scanned from 0 to 3 V at the Pt top electrode. The W bottom electrode was grounded during the DC I−V measurements. Under the positive bias condition, the positively charged Cu ions (Cu^+^) in the Cu_26_Ge_24_Se_50_ reservoir layer drift toward the Ge_30_Se_70_ resistive switching layer, thereby forming conductive Cu filaments in the Ge_30_Se_70_ resistive switching layer through an electrochemical reaction (i.e., a redox process) [44, 45]. Then, the unstable Cu filaments self‐ruptured by diffusive dynamics under the no‐bias condition owing to the lightly alloyed Cu atoms in the Cu_26_Ge_24_Se_50_ reservoir layer. Therefore, the SR‐SOM can perform consecutive threshold switching at V_th, set_ to 1.3 V (i.e., set state of the SR‐SOM), similar to the conventional diffusive memristor (i.e., selector) behavior [46]. Conversely, when the voltage bias was scanned from 0 to ‐3 V, the SR‐SOM exhibited no current flow at the negative bias condition in the red line of Figure 1f, with self‐rectifying characteristics like an ideal diode. This self‐rectifying property was associated with the generation of high resistance in the pure Ge_30_Se_70_ resistive switching layer, where the spontaneously ruptured Cu filaments released Cu ions that subsequently migrated toward the Cu_26_Ge_24_Se_50_ reservoir layer under the applied negative bias (inset illustration (iii) of Figure 1f). Unlike previously reported SOMs, our SR‐SOM does not involve high currents during the negative write process because of its self‐rectifying property and can achieve lower write‐energy consumption in a vertically stacked high‐density cross‐point array. After the negative write process, when the voltage bias was again scanned from 0 to 3 V, the SR‐SOM was abruptly switched at V_th, reset_ of 2.0 V (i.e., reset state of the SR‐SOM), not V_th_ of 1.3 V, as shown in the green line of Figure 1f. Note that the SR‐SOM returned to the set state (i.e., V_th, set_ to 1.3 V) after abrupt threshold switching at a V_th, reset_ of 2.0 V. That is, the SR‐SOM has two V_th_ of 1.3 V (set state) and 2.0 V (reset state) depending on the bias polarity of the write process, being able to readout two resistance states (i.e., high resistance state (HRS) and low resistance state (LRS)) of the SR‐SOM at read voltage of ∼1.6 V. This result indicates that SR‐SOM can perform the dual function of a selector and memory device via a polarity‐induced V_th_ shift by Cu atomic migration in DFM, exhibiting a sufficient RWM of ∼0.7 V. In addition, the RWM and operation voltages of the SR‐SOM can be tuned by controlling the concentration of Cu atoms in the Ge_30_Se_70_ resistive switching layer. Detailed information on the optimized Cu co‐sputtering power of 40 W for maximizing the RWM is provided in the Methods section.

**FIGURE 1:**
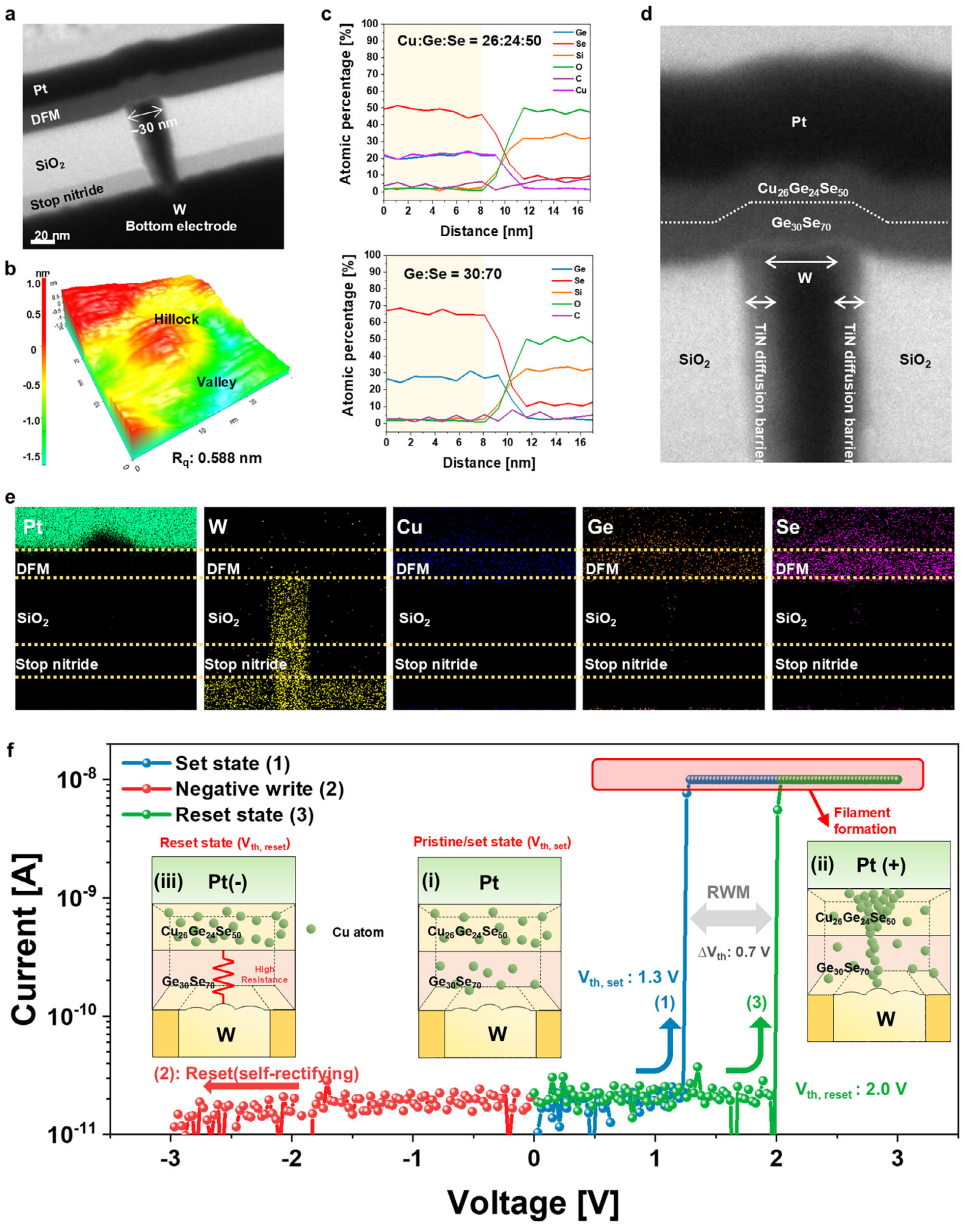
Self‐rectifying selector‐only memory. (a) x‐TEM image of the SR‐SOM. (b) The cropped surface morphology of the W bottom electrode with 30‐nm‐diameter. (c) The atomic concentration of the Ge_30_Se_70_ resistive switching layer and the Cu_26_Ge_24_Se_50_ reservoir layer in DFM. (d) The magnified x‐TEM image of the SR‐SOM. (e) EDS mapping image. (f) DC I‐V curve of the SR‐SOM having self‐rectifying properties and polarity‐induced V_th_ shift.

## 3 Operation Mechanism of the SR‐SOM

The SR‐SOM had V_th_ of 1.3 V at set states (i.e., after positive write process) and V_th_ of 2.0 V at reset states (i.e., after negative write process), confirming that the polarity of the applied write bias directly governs the shift of V_th_ in the SR‐SOM. To elucidate the underlying mechanism of this polarity‐induced V_th_ shift, the atomic migration behavior of the Cu ions was examined using ToF‐SIMS depth profiling as a function of the applied voltage bias history. First, we prepared three samples of 60 × 60 µm^2^ scaled SR‐SOM having the double stacked Cu_26_Ge_24_Se_50_ reservoir layer and Ge_30_Se_70_ resistive switching layer with same thickness of the 30‐nm‐diameter scaled SR‐SOM, as illustrated in Figure S3. In the DC I−V curve of Figure 2a, microscale SR‐SOM conducted threshold switching at V_th, set_ of 1.35 V and V_th, reset_ of 2.03 V similar to those of the nanoscale (30‐nm‐diameter) SR‐SOM regardless of cell diameter. In particular, the self‐rectifying property was observed in the microscale SR‐SOM under negative bias conditions. Note that the off‐current level was drastically increased up to ∼1 µA, at read voltage of 1.6 V owing to the large area, unlike the nanoscale SR‐SOM. By setting the pristine, set, and reset states of each microscale SR‐SOM, the atomic migration of all the elements (i.e., Pt, Cu, Ge, Se, and W) was analyzed using TOF‐SIMS, as shown in Figure 2b–d. In the pristine state, the peak intensity of the Cu ions was located at the interface of the Cu_26_Ge_24_Se_50_ reservoir layer and the Ge_30_Se_70_ resistive switching layer, indicating that the Cu ions were largely diffused into the Ge_30_Se_70_ resistive switching layer by the high sputtering energy supplied during the deposition process, as shown in Figure 2b. After a positive bias was applied to the Pt top electrode (i.e., positive write process), the depth profile of the Cu ions shifted slightly toward the W bottom electrode depending on the direction of the applied electric field, as shown in Figure 2c. Comparing the depth profiles of the pristine and set states, the intensity profiles of the Cu ions were similar, which indicates that SR‐SOM exhibits electroforming‐free properties. After a negative bias was applied (i.e., a negative write process), the depth profile of the Cu ions shifted markedly toward the Pt top electrode, with pronounced accumulation observed in the Cu_26_Ge_24_Se_50_ reservoir layer, as shown in Figure 2d. Unlike other elements, the movement of Cu ions varied significantly depending on the history of the voltage bias. The intensity of Cu ions at the interface region between the DFM and the W bottom electrode (i.e., 40 nm depth) was 15737.05 (a.u.) in the pristine state, increased to 19619.29 (a.u.) in the set state, and decreased again to 7734.95 (a.u.) in the reset state, as shown in Figure 2e. These movements of Cu ions were attributed to the high mobility of Cu ions in chalcogenide materials [47], indicating that the polarity of the write voltage bias could change the concentration of metallic Cu atoms in the Ge_30_Se_70_ resistive switching layer; that is, the larger the Cu quantity, the lower the resistance, and the smaller the Cu quantity, the higher the resistance. This analysis indicates that the V_th_ of the SR‐SOM can be tuned by the polarity‐induced migration of Cu ions in the DFM.

**FIGURE 2:**
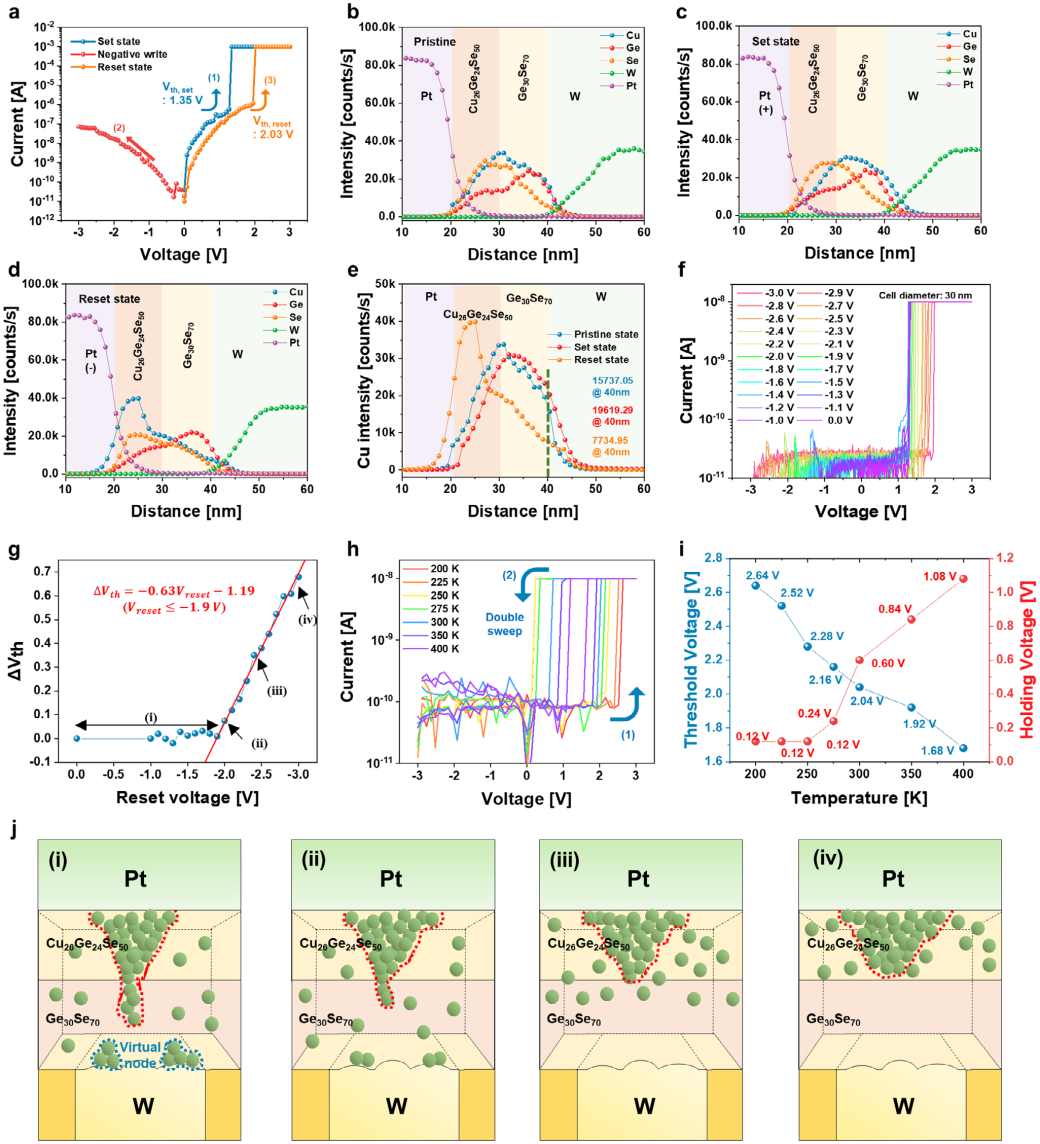
Polarity‐induced V_th_ shift and abrupt threshold switching mechanism of the SR‐SOM. (a) DC I‐V curve of the microscale SR‐SOM. (b–d) SIMS depth profiles of the pristine, set, and reset states, respectively. (e) Atomic migrations of the Cu ions in DFM depending on the history of the applied biases. (f,g) V_th_ modulation of the nanoscale SR‐SOM by increasing the amplitude of the applied negative voltage bias. (h,i) DC I‐V curves of the nanoscale SR‐SOM at a near‐cryogenic temperature of 200 K to a high temperature of 400 K and the variation of the V_th_ and V_hold_. (j) Gradual V_th_ modulation of the nanoscale SR‐SOM via negative voltage‐controlled atomic migration of the Cu ions in DFM.

The high mobility of Cu‐ions, which reflects their rapid electrochemical response to an applied voltage bias, suggests that the extent of Cu‐ion redistribution within the DFM can be modulated by the applied bias. As the electric field strength increased, the extent of Cu‐ion migration within the DFM correspondingly varied, leading to a linearly proportional adjustment in the resistance of the Ge_30_Se_70_ resistive switching layer. Based on these unique Cu‐ion dynamics, V_th_ of the SR‐SOM was tuned by varying the amplitude of the negative voltage bias, as shown in Figure 2f. When the amplitude of the negative voltage bias gradually increased from −1 to −3 V at intervals of 0.1 V, the RWM (i.e., ∆V_th_) did not change until the amplitude of the negative voltage bias reached −1.9 V, but after −2.0 V, the RWM increased linearly from 0 to 0.7 V, as shown in Figure 2g. In general, redox devices operated by the generation and annihilation dynamics of metal‐atom‐based filaments locally accumulate extra metal atoms in the bottom electrode region when continuous resistive switching is performed [48], as illustrated in (i) of Figure 2j. These accumulated metal atoms can act as virtual nodes, which is presumed to be the cause of the phenomenon in which V_th_ did not change even when the reset voltage increased to −1.9 V. Above the reset voltage of −2.0 V, energy is supplied to ionize even the accumulated metal atoms, and the migration of Cu ions proceeds, and V_th_ appears to change gradually, as illustrated in (ii) of Figure 2j. At the point where RWM changes prominently (i.e., the reset voltage was −2.5 V or higher), the Cu ion concentration in the Ge_30_Se_70_ resistive switching layer changes significantly, causing V_th_ to shift (illustration (iii) of Figure 2j), and at the −3.0 V point, the Cu ions in the Ge_30_Se_70_ resistive switching layer nearly disappear (illustration (iv) of Figure 2j). Based on these dynamics, our SR‐SOM exhibits controllable V_th_ modulation through Cu‐ion migration in the DFM, which may provide a basis for future exploration of multilevel‐state programming. This tunability of the V_th_ states may provide a basis for increasing bit‐per‐cell capacity in future high‐density memory applications [49].

The dynamics of mobile Cu ions within the DFM are strongly affected by temperature. Therefore, the formation and spontaneous rupture of conductive Cu filaments are expected to exhibit temperature‐dependent behavior. To further elucidate the abrupt TS mechanism associated with conductive Cu filaments, we measured the double‐sweep DC I−V characteristics over a wide temperature range of 200–400 K, as shown in Figure 2h. In the double‐swept DC I−V measurements, both V_th_ and V_hold_ were extracted to assess the ease of the self‐annihilation response of the conductive filaments. At 200 K, the SR‐SOM exhibited an abrupt transition from the HRS to the LRS at V_th_ = 2.64 V during the forward voltage sweep from −3 to 3 V. As the temperature increased to 400 K, V_th_ gradually decreased to 1.68 V, as summarized in Figure 2i. Here, V_th_ represents the dynamic onset voltage at which an electrically connected Cu filament is established within the voltage sweep. Filament‐formation involves the electrochemical oxidation, field‐assisted Cu‐ion migration, reduction, and subsequent nucleation and growth of a metallic conductive filament within the resistive switching layer [50]. Because the associated ion‐transport and nucleation processes are thermally activated, the characteristic filament‐formation time decreases with increasing temperature and exhibits a strong dependence on both the applied voltage and temperature [51, 52]. Consequently, under an identical voltage‐sweep condition, elevated temperature enables an electrically connected Cu filament to form earlier in the sweep and at a lower applied voltage, accounting for the observed decrease in V_th_. In the subsequent reverse sweep from 3 to −3 V, the SR‐SOM returned from the LRS to the HRS at V_hold_ = 0.12 V at 200 K. With increasing temperature, V_hold_ increased to 1.08 V at 400 K, as shown in Figure 2i. Notably, V_hold_ remained close to zero below 300 K, indicating that the conductive filament remained continuous until the applied bias was nearly removed. In contrast to V_th_, V_hold_ represents the minimum sustaining bias required to preserve the electrical continuity of an already‐formed weak filament. Once the voltage decreases below V_hold_, the filament spontaneously relaxes and the device returns to the HRS. Filament stability is governed by their morphology, the surrounding matrix, the applied bias, and temperature; at the nanoscale Ag and Cu filaments can spontaneously disintegrate through atomic surface diffusion driven by surface‐energy minimization [53, 54]. The surface diffusion coefficient follows the thermally activated relation:

(1) $$D_{s}=D_{s}^{\prime }\;\exp\left(-\frac{Q_{s}}{k_{B}T}\right)$$

where *D_s_* is the surface‐diffusion coefficient, $D_{s}^{\prime }$ is the pre‐exponential factor, *Q_s_* is the surface‐diffusion barrier, *k*_B_ is the Boltzmann constant, and *T* is the absolute temperature [54]. As a result, the rate of Cu‐atom redistribution at the filament surface increases at elevated temperatures. As the applied voltage decreases during the reverse sweep, the decreasing electric field progressively weakens the field‐assisted Cu transport that helps sustain the narrowest region of the filament, whereas thermally activated atomic redistribution continues to destabilize the filament. V_hold_ can therefore be interpreted as a kinetic crossover voltage at which field‐assisted filament maintenance is no longer sufficient to offset spontaneous filament dissolution. At elevated temperatures, the accelerated dissolution kinetics require a larger sustaining bias to preserve electrical continuity. Consequently, the filament ruptures at a higher voltage during the reverse sweep, accounting for the increase in V_hold_ from 0.12 V at 200 K to 1.08 V at 400 K. These opposing trends of V_th_ and V_hold_ ultimately imply that the abrupt TS phenomenon of SR‐SOM is dominated by the generation and rupture of conductive Cu filaments. Thus, the abrupt threshold switching of the SR‐SOM was associated with the generation of conductive Cu filaments in the DFM, unlike other SOM operated by the OTS mechanism.

## 4 Electrical Performance of the Self‐Rectifying Selector‐Only Memory

To reiterate, the SR‐SOM performed abrupt threshold switching through the formation of conductive Cu filaments within the DFM and suppressed the current flow in the negative‐bias region owing to its intrinsic self‐rectifying behavior. Because of the filamentary switching mechanism and self‐rectifying properties, the nanoscale SR‐SOM with a 30‐nm‐diameter could achieve superior electrical performance, such as high selectivity, cell‐diameter independent V_th_, high write/erase endurance, and specifically, drift‐free properties, as shown in Figure 3. The filamentary switched SR‐SOM has a high selectivity of ∼10^5^ at a compliance current level of 1 µA without variation of V_th_, as shown in Figure 3a. In addition, the SR‐SOM performed threshold switching at V_th, set_ to 1.3 V, and V_th, reset_ to 2.0 V for a diameter of 30‐ to 618‐nm‐diameter, as shown in Figure 3b. Therefore, the RWM of the nanoscale SR‐SOM could be retained independently of the cell diameter, as shown in Figure 3c. Because of the filamentary switching mechanism and the use of the Ge_30_Se_70_ resistive switching layer having a high energy bandgap of 1.29 eV, the SR‐SOM exhibited an ultralow leakage current below 0.1 nA and an identical threshold switching voltage regardless of compliance current levels and cell diameters. In addition, the electrical performance of the memory devices should be robust at high temperatures memory devices of Vthegative bias region due to self‐rectifying properties. To evaluate the fluctuation of V_th_ of the SR‐SOM at high temperatures of 85°C, the DC I−V curves were obtained at intervals of 30 min for 6 h, as shown in Figure 3d, and the corresponding time‐dependent V_th_ is shown in Figure 3e. Initially, the SR‐SOM had V_th, set_ of 1.30 V and V_th, reset_ of 2.04 V, but V_th, set_ and V_th, reset_ were slightly decreased from 1.30 to 1.13 V and from 2.04 to 1.86 V, respectively. However, V_th, set_ and V_th, reset_ were saturated at ∼1.1 and ∼1.8 V with increasing time, respectively. This reduction in the V_th_ of the SR‐SOM was associated with an increase in the diffusion of Cu ions into the Ge_30_Se_70_ resistive switching layer from the Cu_26_Ge_24_Se_50_ reservoir layer at high temperatures. Although the reduction of V_th_ has occurred at a high temperature of 85°C, the RWM (i.e., ∆V_th_) of the SR‐SOM remained nearly constant at ∼0.7 V over time, as shown in Figure 3f. In addition, normal quantile plot of V_th, set_ and V_th, reset_ revealed a minimum RWM of ∼0.52 V as shown in the inset of Figure 3f. In other words, the resistance state of the SR‐SOM can still be distinguished at V_read_ of 1.5 V under high‐temperature conditions. To further assess the retention behavior, direct resistance‐retention measurements at 85°C for 10^5^ s and an Arrhenius plot of retention failure time obtained from accelerated measurements up to 150°C are provided in Figure S4.

**FIGURE 3:**
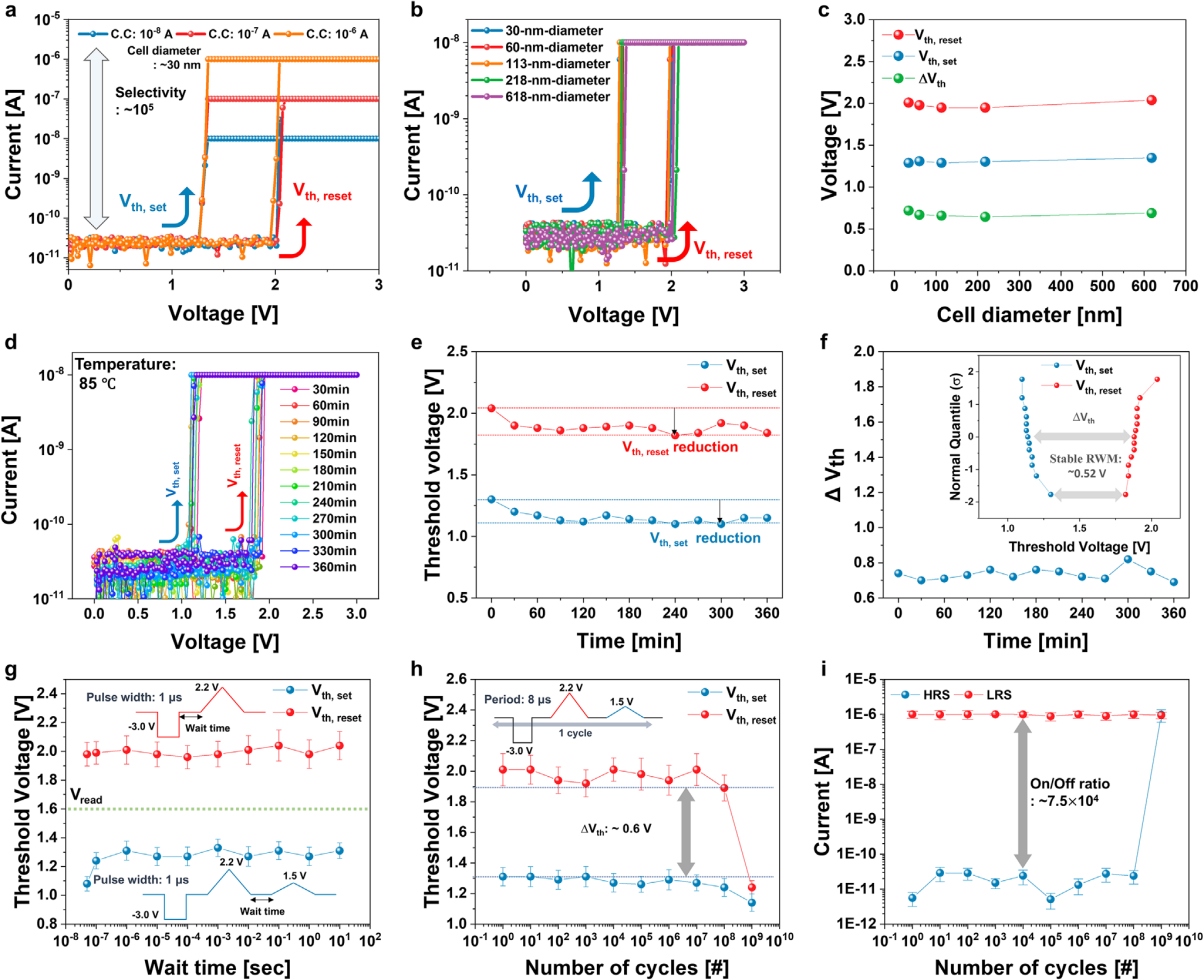
Electrical robustness and drift‐free properties of the nanoscale SR‐SOM. (a) DC I‐V curves of the SR‐SOM depending on the compliance current level, showing high selectivity of ∼10^5^. (b,c) V_th_ and RWM independency of the SR‐SOM depending on the cell diameter from 30 to 618 nm. (d,e) DC I‐V curves and slight V_th_ reduction with times at a temperature of 85°C. f) Near‐constant RWM of ∼0.7 V and stable RWM achievements of the nanoscale SR‐SOM. g) Drift‐free properties of the nanoscale SR‐SOM. (h,i) A Highly reliable nanoscale SR‐SOM having a high endurance of ∼10^8^. To measure V_th, set_, V_th, reset_, and currents (i.e., on‐ and off‐current) simultaneously, a square pulse with an amplitude of −3.0 V and a pulse width of 1 µs for reset process, a triangular pulse with an amplitude of 2.2 V and a pulse width of 2 µs for write process, and a triangular pulse with an amplitude of 1.5 V and a pulse width of 2 µs for read process were sequentially inputted to nanoscale SR‐SOM as a 1 cycle pulse. Here, the intervals of each pulse were 1 us, so the total period of 1 cycle pulse was 8 µs.

For the non‐filamentary SOM operated by the OTS mechanism, as summarized in Table 1, the drift phenomenon inherently occurs in consecutive programming processes by the atomic rearrangement of pure chalcogenide materials [36, 37, 38]. Therefore, it can result in retention failure of the stored data as well as delay issues during the operation of high‐density cross‐point arrays. Unlike the OTS‐based SOM, the filamentary switched SR‐SOM could achieve drift‐free properties after a waiting time of ∼100 ns, as shown in Figure 3g, which means that, assuming a read voltage of 1.6 V, the SR‐SOM exhibited high retention time at room temperature. The drift‐free properties of SR‐SOM were due to the fast rupture time (i.e., holding time) of the conductive Cu filaments formed within the DFM, as shown in Figure S5. A triangular voltage pulse was employed to evaluate the V_th_ response during the pulse operation. When a triangular pulse with an amplitude of 2.2 V was applied following a negative reset pulse with an amplitude of −3 V, the SR‐SOM showed an abrupt change from HRS to LRS at V_th, reset_ of 2.0 V, and subsequently returned from the LRS to the HRS at V_hold_ of 0.6 V, as shown in Figure S5a. Here, the pulse width of all voltage pulses was 1 µs. Notably, during the write process, the switching speed and holding time of the SR‐SOM were ∼40 and ∼45 ns, respectively, as shown in Figure S5b,c. In addition, in the read process, the SR‐SOM performed threshold switching and spontaneous rupture in only ∼20 ns, as shown in Figure S5d–f. Based on these drift‐free properties enabled by the rapid rupture of conductive Cu filaments, SR‐SOM can operate without drift‐induced delay time (i.e., wait time) in high‐density cross‐point arrays.

Furthermore, unlike conventional filamentary switching memristors, such as resistive random‐access memory (ReRAM) and conductive‐bridge random‐access memory (CBRAM), which suffer from high reset current and reliability degradation [55, 56], the SR‐SOM suppresses current flow under negative bias owing to its self‐rectifying properties. This feature minimizes the electrically induced thermal stress, thereby achieving a high write/erase endurance (∼10^8^), comparable to that of OTS‐based SOM, as shown in Figure 3h,i [57]. Here, the minimal RWM (i.e., ∆V_th_) of ∼0.6 V and on/off ratio of ∼7.5 × 10^4^ were retained with write/erase cycles, respectively. To examine whether repeated switching induced Cu out‐diffusion into the surrounding dielectric, cross‐sectional STEM–EDS analysis was performed on an additional 200‐nm SR‐SOM device subjected to 10^8^ write/erase cycles. No discernible Cu accumulation was observed in the adjacent SiO_2_, supporting the predominant confinement of Cu within the intended active stack under the investigated endurance conditions is shown in Figure S6. To further evaluate the array‐level feasibility of the SR‐SOM, we estimated the readout margin under a half‐bias scheme using a current‐sensing‐based analysis of the measured read currents [58]. As shown in Figure S7, the readout margin remained above 90% up to a 2^13^×2^13^ crossbar array, corresponding to ∼67 Mbit, indicating effective suppression of sneak‐path current.

## 5 Combinational Logic Design via the Self‐Rectifying Selector‐only Memory‐Based Logic‐In‐Memory

A logic‐in‐memory‐enabled computing architecture can significantly reduce the overload of the CPU or other processing units, thereby enhancing the energy efficiency of the total computing system. We demonstrate 16 Boolean logic gates and a full adder using one or more SR‐SOMs via sequential logic schemes. Specifically, the input and output were considered as the voltage and resistance in sequential logic, respectively, unlike stateful logic [59]. For the 14 Boolean logic gates, except for exclusive OR (XOR) and exclusive NOR (XNOR), we referred to previously reported sequential logic [60], demonstrating AND, NAND, OR, NOR, and other logic gates, as shown in Figure 4a and Figure S8. Here, ‘T,’ ‘B,’ ‘High,’ and ‘Low’ represent the top electrode, the bottom electrode, 3 V, and 0 V in the sequential pulse table, respectively. In addition, ‘X’ and ‘Y’ denote operands for Boolean logic gates. For instance, in the case of the AND logic gate that operated with ‘X = 0’ and ‘Y = 0’, the 3 V and 0 V with a pulse width of 100 µs were applied to the Pt top electrode and W bottom electrode in Cycle 1, respectively. In cycle 2, 0 and 3 V were applied to the Pt top electrode and W bottom electrode, respectively. Finally, 0 and 3 V were applied to the Pt top electrode and W bottom electrode in cycle 3, respectively. Therefore, the SR‐SOM exhibits HRS currents when a read voltage of 1.5 V was applied. Hence, the SR‐SOM presented a ‘Low’ state at ‘X = 0’ and ‘Y = 0’ in the AND logic gate. In this way, when the AND logic gate was operated with ‘X = 1’ and ‘Y = 0’, ‘X = 0’ and ‘Y = 1’, and ‘X = 1’ and ‘Y = 1’, the SR‐SOM showed a ‘Low,’ ‘Low,’ and ‘High’ states, respectively, as shown in the right pulse diagram of the sequential pulse table. Note that a compliance current was set to 10 nA, and ‘T‐B’ represents the difference of applied voltage pulses at the top and the bottom electrodes.

**FIGURE 4:**
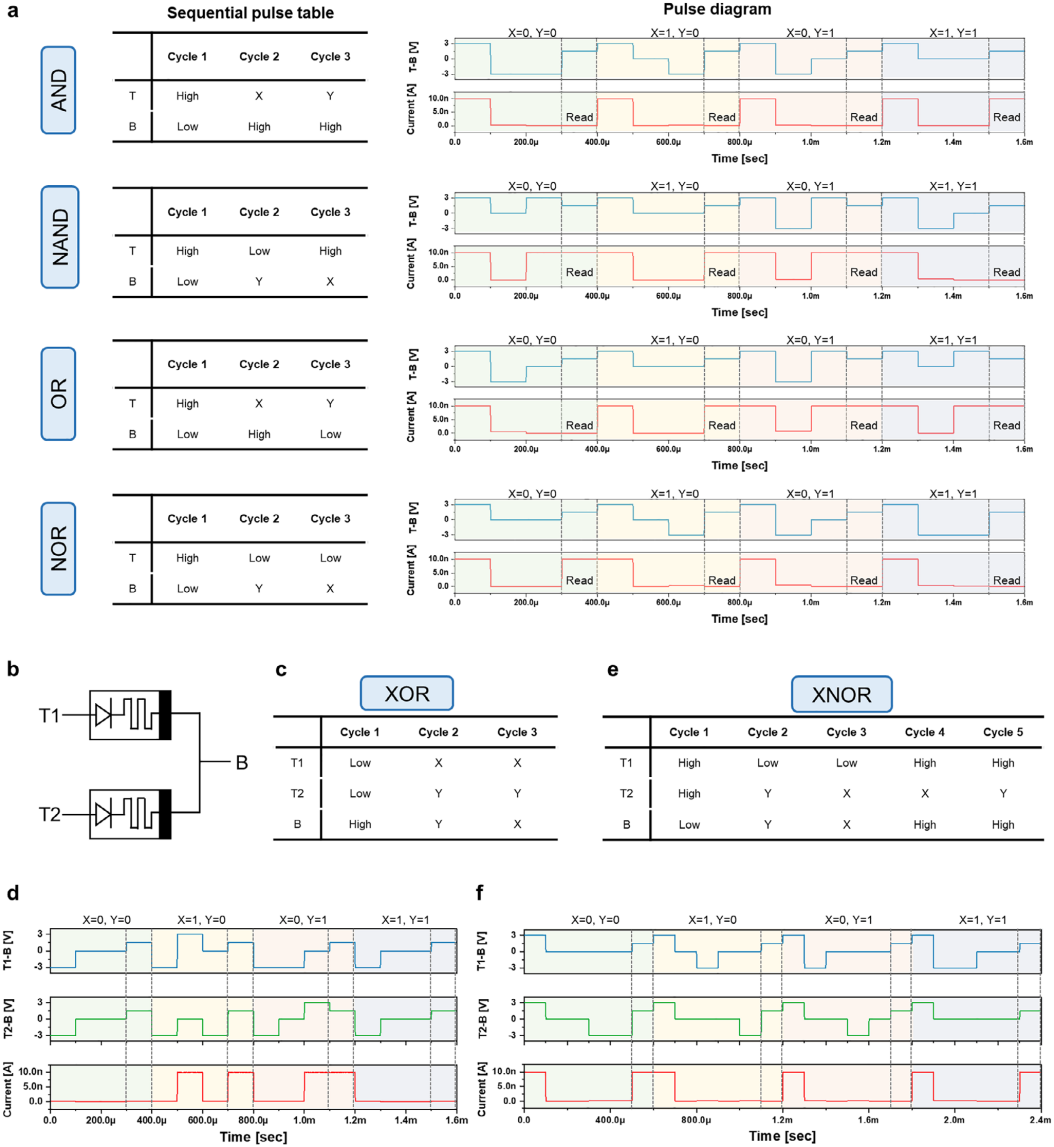
Implementation of the 16 Boolean logic gates via the SR‐SOM‐based logic‐in‐memory. (a) Sequential pulse table and pulse diagram of the representative 4 Boolean logic gates (i.e., AND, NAND, OR, and NOR) via a single SR‐SOM using sequential logic schemes. The other 12 Boolean logic gates (i.e., IMP, NIMP, RIMP, RNIMP, and etc.) were described in Figure S8. (b) Parallelized two SR‐SOMs with a global bottom electrode for operating complex XOR and XNOR logic gates. (c–f) Designed a sequential pulse table and pulse diagram of the XOR and XNOR using two SR‐SOMs.

To implement complex combinational logic such as XOR and XNOR, two parallel SR‐SOM with a global bottom electrode were used, as shown in Figure 4b. Notably, the two SR‐SOM‐based memory arrays with a global bottom electrode can flow high current during the read process if only one of the two is in a ‘High’ state. Through this approach, XOR and XNOR logic gates were designed with a combination of negated implication (NIMP) AND and NOR reverse negated implication (RNIMP) logic gates and a combination of AND and NOR logic gates, respectively, as shown in the algebraic function of the Boolean logic table in Table S1. A sequential pulse table for the XOR logic gate, designed by combining the NIMP and RNIMP logic gates, is shown in Figure 4c. When the voltage cycles were inputted sequentially following a sequential pulse table of the XOR gate, the resistance of the SR‐SOM exhibited ‘Low’, ‘High’, ‘High,’ and ‘Low’ at read process according to operands of ‘X = 0’ and ‘Y = 0’, ‘X = 1’ and ‘Y = 0’, ‘X = 0’ and ‘Y = 1’, and ‘X = 1’ and ‘Y = 1’, respectively (Figure 4d). Similarly, when the voltage cycles were inputted sequentially following a sequential pulse table of the XNOR gate designed by combining AND and NOR logic gates in Figure 4e, the resistance of the SR‐SOM presented ‘High’, ‘Low’, ‘Low’, and ‘High’ at read process depending on the combination of two operands, as shown in Figure 4f. Thus, the SR‐SOM‐based logic‐in‐memory approach successfully demonstrated all 16 Boolean logic gates within five cycles.

Using the 16 Boolean logics implemented by the SR‐SOM‐based logic‐in‐memory, a full adder was designed by combining the three SR‐SOMs, as shown in Figure 5a. In particular, we divided the truth table of the full adder consisting of three inputs (X, Y, and carry‐in (C_in_)) and two outputs (sum and carry‐out) into two parts, as shown in Table S2: In the case of ‘X = 0’ (i.e., the part of green color), the sum and carry‐out represent XOR and AND logic gate depending on the combination of Y and C_in_ operands. In addition, in the case of ‘X = 1’ (i.e., the part of blue color), the sum and carry‐out exhibit XNOR and OR logic gate depending on the combination of Y and C_in_ operands. That is, the ‘X’ operands can be considered as an ‘Enable’ signal. As mentioned above, we demonstrated XOR and XNOR logic gates using two SR‐SOM‐based arrays, and implemented AND and OR logic gates using a single SR‐SOM. Through these approaches, the sequential pulse tables of full adder in the case of ‘X = 0’ and ‘X = 1’ were designed, as shown in Figure 5b,c. Thereby, a stable full adder having three inputs and two outputs (i.e., sum and carry‐out) was successfully designed using three SR‐SOMs (i.e., two SR‐SOMs for sum and a single SR‐SOM for carry‐out), as shown in Figure 5d,e. To quantitatively evaluate the operating energy of the proposed logic‐in‐memory operation, the energy consumption for each pulse sequence was calculated for all input combinations. As summarized in Table S1, the 16 Boolean logic functions exhibit worst‐case energy consumption of 4.5–7.51 pJ per operation. For the three‐SR‐SOM full adder, the total operation energy is 7.52 pJ for X = 0 and 15.0 pJ for X = 1, corresponding to 3 and 5 cycles, respectively. A benchmark comparison with CMOS‐based and emerging non‐volatile‐memory‐based 1‐bit full adders is provided in Table S3. By demonstrating all 16 Boolean logic functions and a full‐adder operation using SR‐SOMs, we verify the feasibility of compact logic‐in‐memory implementations that integrate data storage and logic processing within SR‐SOM cells while achieving pJ‐level operating energy.

**FIGURE 5:**
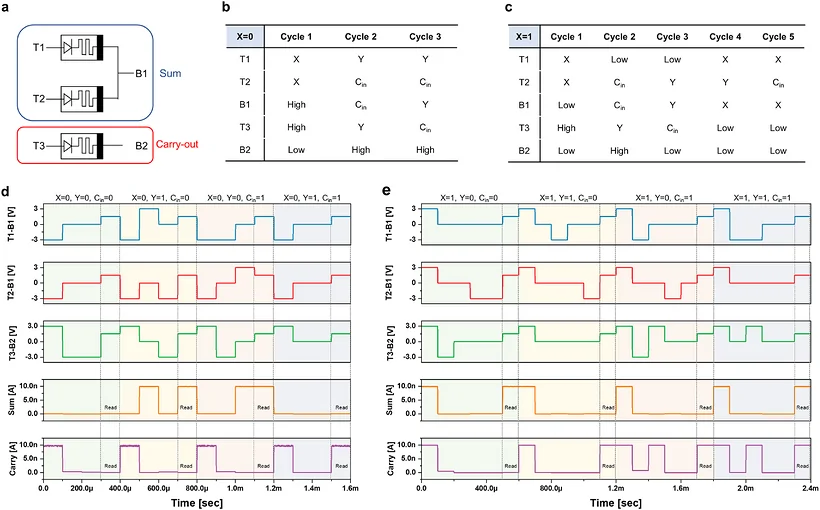
Design of full adder via three SR‐SOMs‐based logic‐in‐memory. (a) Parallelized two SR‐SOMs with a global bottom electrode and a single SR‐SOM for implementing a sum and carry‐out operation. (b,c) Sequential logic pulse table in the case of ‘X = 0’ and ‘X = 1’, respectively. (d,e) Pulse diagrams of the full adder using three SR‐SOMs.

## 6 Conclusions

In this study, we developed a novel SR‐SOM based on a bilayer‐stacked DFM comprising a Cu_26_Ge_24_Se_50_ reservoir layer and Ge_30_Se_70_ resistive switching layer operating through the formation and rupture of conductive Cu filaments. The SR‐SOM exhibited polarity‐induced V_th_ modulation arising from Cu‐ion migration within the DFM, thereby enabling the simultaneous realization of selector and memory functionalities. Compared with previously reported OTS‐based SOM, the filamentary‐switched SR‐SOM demonstrated several key advantages, including being electroforming‐free, high selectivity (∼10^5^), and fast switching speed (<40 ns). Moreover, the SR‐SOM presented a high write/erase endurance of ∼10^8^ comparable to that of the nonfilamentary SOM, by lowering the electrically induced thermal stress during the negative write process. Importantly, the intrinsic drift‐free behavior of the SR‐SOM could be achieved by the ultrafast rupturing (i.e., <20 ns) of the Cu filaments within the DFM, supporting its potential applicability to high‐density CXL memory systems. Furthermore, we implemented all 16 Boolean logic functions and a full adder operation using SR‐SOM devices through a logic‐in‐memory scheme, thereby verifying the feasibility of compact SR‐SOM‐based logic‐in‐memory implementations. Overall, these results suggest that SR‐SOMs provide a promising route toward compact, self‐rectifying memory cells that combine polarity‐tunable V_th_‐based memory functionality with logic‐in‐memory capability, offering potential for high‐density storage and memory‐centric computing systems.

## 7 Methods

### 7.1 Fabrication of the Nanoscale SR‐SOM

A 20‐nm‐thick Si_3_N_4_ and 50‐nm‐thick SiO_2_ layer were sequentially deposited on a 12‐inch W wafer by chemical vapor deposition (CVD), and 30‐nm‐diameter nanoholes were patterned by photolithography and dry etching. Plug‐type W bottom electrodes with a thickness of 70 nm were fabricated by sequentially depositing a TiN diffusion barrier and W film on the nanoholes, followed by the CMP process. Then, a 200 µL of a photoresist (AZ5214E) was spin‐coated on the plug‐type W patterned coupon wafer with a size of 1.0 × 1.5 cm^2^ at 5000 rpm for 30 sec and soft baked at 120°C for 100 sec to fabricate the active cell region of the SR‐SOM. In addition, a photomask with 60 × 60 µm^2^ patterns was aligned on the plug‐type W‐patterned wafer and exposed to UV light with a beam intensity of 20 mW/cm^2^ for 4 sec. The exposed photoresist was developed for 70 s using a developer (AZ300MIF). The 10‐nm‐thick Ge_30_Se_70_ resistive switching layer was deposited on the plug‐type W bottom electrode with dimensions of 60 × 60 µm^2^ by sputtering the GeSe target at an RF power of 30 W under an Ar flow of 40 sccm. A 10‐nm‐thick Cu_26_Ge_24_Se_50_ reservoir layer was deposited by co‐sputtering GeSe at an RF power of 30 W and Cu targets at an RF power of 40 W under an Ar flow of 40 sccm. A 30‐nm‐thick Pt top electrode was sputtered using a Pt target at a DC power of 30 W under an Ar flow of 30 sccm. Finally, the SR‐SOM was patterned using a lift‐off process with an acetone, methanol, and DI water rinse. Therefore, the SR‐SOM was completely fabricated with a vertical structure consisting of a 30‐nm‐diameter W bottom electrode, Ge_30_Se_70_ resistive switching layer, Cu_26_Ge_24_Se_50_ reservoir layer, and Pt top electrode.

### 7.2 Fabrication of the Microscale SR‐SOM for Analyzing the Polarity‐Induced V_th_ Shift Mechanism

The microscale SR‐SOM with a size of 60 × 60 µm^2^ was fabricated to conduct SIMS analysis. A 100‐nm‐thick SiO_2_ film was grown on a Si substrate via dry oxidation. Then, a 20‐nm‐thick W film was then deposited on a 100‐nm‐thick SiO_2_ film by sputtering the W target at a DC power of 40 W under an Ar flow of 30 sccm. A 10‐nm‐thick Ge_30_Se_70_ resistive switching layer and a 10‐nm‐thick Cu_26_Ge_24_Se_50_ reservoir layer were sequentially deposited on the W surface as the bottom electrode while screening the lateral regions using a thermal tape. A similar photolithography process was conducted to produce square‐shaped 60 × 60 µm^2^ photoresist patterns. Subsequently, a 20‐nm‐thick Pt top electrode was sputtered using a Pt target at a DC power of 30 W under an Ar flow of 30 sccm. Finally, the microscale SR‐SOM was fabricated using a liftoff process with acetone, methanol, and DI water rinses.

### 7.3 Control of V_th_, RWM, and the Operation Voltages of the SR‐SOM

The V_th_ and RWM of the SR‐SOM could be varied by precisely controlling the Cu co‐sputtering power, as shown in Figure S9a. When the Cu‐doped GeSe reservoir layer was deposited by co‐sputtering, the Cu atoms largely diffused into the Ge_30_Se_70_ resistive switching layer as the Cu co‐sputtering power was increased. That is, the resistance of the Ge_30_Se_70_ resistive switching layer decreased as the concentration of diffused Cu atoms increased. Thereby, in the pristine and set states, the V_th, set_ was decreased slightly from 1.68 to 0.60 V when the Cu co‐sputtering power was increased from 20 to 80 watts at intervals of 20 watts, as shown in Figure S9b. Similarly, the V_th, reset_ was decreased from 2.19 to 1.00 V when the Cu sputtering power increased, indicating that the number of doped Cu atoms increased in the bilayer‐stacked DFM. Through this approach, the RWM (i.e., ∆V_th_) and operation voltages of the SR‐SOM can be easily tuned by varying the concentration of Cu atoms in the Ge_30_Se_70_ resistive switching layer. Here, we selected a Cu co‐sputtering power of 40 watts to achieve the maximum RWM.

### 7.4 Material Characterization

Cross‐sectional transmission electron microscopy and energy‐dispersive X‐ray spectroscopy (TEM and EDS: NEO ARM) of the SR‐SOM were conducted at an acceleration voltage of 200 kV. The surface morphology of the plug‐type W bottom electrode, with a diameter of approximately 30 nm, was examined using atomic force microscopy (AFM, XE7). The atomic composition ratios of the Cu_26_Ge_24_Se_50_ reservoir and Ge_30_Se_70_ resistive switching layers were investigated using Auger electron spectroscopy depth profiles (AES, PHI 700 Xi). In addition, time‐of‐flight secondary ion mass spectrometry (ToF‐SIMS, TOF.SIMS 5) depth profile measurements were performed to analyze the atomic migration of Cu ions in the DFM, depending on the history of the applied voltage biases (i.e., pristine, set, and reset states). The energy bandgaps of the Cu_26_Ge_24_Se_50_ and Ge_30_Se_70_ resistive switching layers were analyzed using UV–vis spectroscopy (VARIAN Cary 5000).

### 7.5 Electrical Characterization

The current‐voltage (I−V) characteristics of the SR‐SOM were assessed using an Agilent B2902A semiconductor parameter analyzer. The write and erase endurance cycles, drift‐free properties, V_th_ variations, and switching speed of the SR‐SOM were evaluated using a Keithley 4200 semiconductor parameter analyzer. The SR‐SOM was placed on a hot chuck controlled by a TAS 1000 temperature controller to measure the DC I−V curve at temperatures ranging from 300 to 400 K. In addition, the DC I−V curve at near‐cryogenic temperatures was measured from 200 to 275 K using a Lakeshore CPX cryogenic probe station under vacuum.

### 7.6 Logic‐In‐Memory Characterization

Sixteen Boolean logic gates and full adders were analyzed using an Agilent B2902A semiconductor parameter analyzer. In Keysight B2902A quick IV measurement software, the pulses corresponding to the sequential pulse table of each Boolean logic were set in the list table. The currents were then read in real time using only one channel. For XOR and XNOR logic using two SR‐SOMs with global bottom electrodes, two channels of an Agilent B2902A semiconductor parameter analyzer were used to apply voltage pulses corresponding to the sequential pulse tables of XOR and XNOR. In addition, in the case of the full adder using three SR‐SOMs, the sum and carry‐out currents were sequentially read owing to the limitations of the two channels.

## Conflicts of Interest

The authors declare no conflicts of interest.

## Supporting information




**Supporting File**: advs77423‐sup‐0001‐SuppMat.docx.

## Data Availability

The data that support the findings of this study are available from the corresponding author upon reasonable request.
