# Supplementary material for: Electroforming‐Free, Self‐Rectifying Selector‐Only Memory With Diffusive Cu‐Ion Dynamics for Logic‐In‐Memory Computing
Source: Adv Sci (Weinh). 2026 Aug 25;13(58):e77423. doi: 10.1002/advs.77423 (PMC13505582; doi:10.1002/advs.77423)
Supplement: Supplementary file 1 [file ADVS-13-e77423-s001.docx]

Supporting Information

**Electroforming-free, Self-Rectifying Selector-Only Memory with Diffusive Cu-ion Dynamics for Logic-In-Memory Computing**

*Jae-Kyeong Kim^*^, Dae-Seong Woo^*^_,_ Min-Jong Han, Hong-Gwang Seo, Gwang-Ho Park, In-Taek Woo, Jea-Gun Park^**^, and Jinsub Park^**^*

**Figure S1.** Tauc plots for calculating the optical energy bandgap of a) the 10-nm-thick Ge_30_Se_70_ resistive switching layer and b) the 10-nm-thick Cu_26_Ge_24_Se_50_ reservoir layer

**Figure S2.** UPS-based band alignment and schematic biased energy band diagrams of our SR-SOM. (a–d) UPS secondary-electron cutoff spectra of Pt, CuGeSe, GeSe, and W. (e) Energy-level alignment constructed from the extracted work functions and optical bandgaps. (f,g) Schematic band diagrams under positive and negative bias, respectively.

The extracted work functions are 4.7 eV for Pt, 4.6 eV for CuGeSe, 4.5 eV for GeSe, and 5.0 eV for W, while the optical bandgaps of CuGeSe and GeSe are 0.99 and 1.29 eV, respectively. Since both CuGeSe and GeSe can be regarded as p-type-like semiconducting layers, the metal/semiconductor contact were assessed in terms of the hole-injection barrier. From the GeSe work function (4.5 eV) and valence-band position (E_F_ − E_V_ ≈ 0.30 eV), the ionization energy of GeSe was estimated to be ∼4.80 eV. Because this value is lower than the work function of W (5.0 eV), the ideal hole-injection barrier at the GeSe/W interface is expected to be negligible. Likewise, from the CuGeSe work function (4.6 eV) and valence-band position (E_F_ − E_V_ ≈ 0.17 eV), the ionization energy of CuGeSe was estimated to be ∼4.77 eV, yielding an ideal hole-injection barrier of ~0.07 eV at the Pt/CuGeSe interface. These small ideal barriers suggest that carrier injection at the metal/chalcogenide contacts is unlikely to be the dominant origin of the observed self-rectification.

The UPS-based band alignment, together with the resistivity contrast between the two chalcogenide layers, indicates an electronically asymmetric p–p isotype bilayer, in which GeSe serves as the wider-gap and highly resistive switching layer, whereas CuGeSe acts as the Cu-ion reservoir.

This interpretation is consistent with the apparent volume resistivity values extracted from the off-state I–V characteristics of Pt/GeSe/W and Pt/CuGeSe/W reference stacks. The apparent resistivity of GeSe (~8.78 × 10^7^ Ω·m) is approximately 5.0 × 10^2^ times higher than that of CuGeSe (~1.75 × 10^5^ Ω·m). Under positive bias on Pt, Cu ions drift from the CuGeSe reservoir toward the GeSe layer, promoting conductive filament formation and abrupt threshold switching. Under negative bias, the field direction is reversed, and Cu ions drift back toward the CuGeSe reservoir layer, leaving the GeSe layer relatively Cu-depleted and highly resistive. As a result, most of the potential drop develops across the Cu-depleted GeSe region under reverse bias, thereby suppressing reverse current. In this sense, the self-rectifying behavior of the SR-SOM can be mainly attributed to the bias-induced resistance asymmetry of the CuGeSe/GeSe bilayer.

**Figure S3.** Fabricated microscale SR-SOM for analyzing atomic migration of the Cu ions in DFM

**Figure S4.** High-temperature retention characteristics of the SR-SOM. (a) HRS and LRS resistance measurement at 85 ℃ for 10^5^ s. (b) Arrhenius plot of the retention failure time.

To directly assess retention characteristics under elevated temperature, we monitored the resistance of the two programmed V_th_ states under repeated read operations at V_read_ of 1.5 V at 85 °C for 10^5^ s. In our SR-SOM, the programmed low-V_th_ set state is read as the LRS, whereas the programmed high-V_th_ reset state is read as the HRS under the read condition. Therefore, the resistance separation measured at V_read_ of 1.5 V directly reflects the retention of the readable memory states. The resistance measurement data show that the LRS and HRS remain clearly distinguishable throughout 10^5^ s at 85 °C. In addition, to further quantify the thermal stability of the programmed state, retention measurements were additionally carried out at elevated temperatures up to 150 °C. Due to the decrease of V_th,reset_ with increasing temperature, as discussed in the main Figure 2h, 150 °C was selected as the highest accelerated-test temperature at which the programmed read states could still be meaningfully discriminated with respect to the read voltage of 1.5 V. For the Arrhenius plot of the retention failure time, we monitored the temporal evolution of V_th,reset_ and defined the retention failure time as the time at which V_th,reset_ decreased to V_read_ = 1.5 V. This criterion directly corresponds to the practical loss of the programmed read margin, since the high-V_th_ reset state can no longer be read as HRS once V_th,reset_ ≤ V_read_. The Arrhenius plot of the extracted failure times yields an effective activation energy of ∼1.39 eV and an extrapolated 10-year retention temperature of ∼52 ℃.

**Figure S5.** Fast switching speed and rupturing time of the SR-SOM

 Fig S6. Cross-sectional STEM-EDS elemental maps of Pt, Cu, Si, and W obtained from separately prepared SR-SOM devices in the set, reset and after endurance stress.

To directly investigate possible lateral Cu out-diffusion into the surrounding SiO_2_, site-specific cross-sectional STEM–EDS analyses were performed on individually prepared 200-nm SR-SOM devices. Individual devices were programmed into the SET state using +3 V or the RESET state using −3 V, while an additional device was subjected to 10^8^ write/erase cycles. The buried W BEC of each device was subsequently located, and cross-sectional FIB lamella were prepared through the corresponding electrically active region for STEM–EDS elemental mapping. The Si and W maps identify the surrounding SiO_2_ dielectric and the embedded W BEC, respectively. Under the SET, RESET, and endurance-stressed conditions, the Cu signals remained predominantly confined to the chalcogenide active-stack region, with no discernible localized Cu accumulation in the adjacent SiO₂, including along the lateral perimeter of the W BEC. Notably, no discernible accumulation was observed even after 10^8^ write/erase cycles. Together with the pristine-state elemental mapping presented in Figure 1e, these results enable a structural comparison among pristine, electrically programmed, and endurance-stressed devices. Although Cu migration below the spatial resolution and detection sensitivity of STEM–EDS cannot be completely excluded, the present observations support the predominant confinement of Cu within the intended Pt/Cu_26_Ge_24_Se_50_/Ge_30_Se_70_/W active region under the investigated conditions.

**Figure S7.** Readout-margin analysis of the SR-SOM crossbar array under a half-bias scheme. (a) Applied voltage distribution of fully-, half-, and un-selected cells in crossbar array under a half-bias scheme. (b) Calculated readout margin as a function of the number of word lines.

Under the half-bias scheme, the fully-selected cell received V_read_, whereas the half-selected cells received V_read_/2. To provide a conservative benchmark, the fully-selected cell was placed in the HRS state (corresponding to the high-V_th_ state), whereas the half-selected cells were assumed to be in the LRS state (corresponding to the low-V_th_ state), consistent with prior half-bias readout-margin analyses of 1S1R crossbar arrays. Because the SR-SOM is a selector-only memory that stores information in the V_th_ state, both the fully-selected HRS cell and half-selected LRS cells exhibit similarly low I_read_ under a half bias scheme, analogous to 1S1R crossbar arrays operation. The sensing current was defined as I_sensing_ = I_HRS_ + total I_leakage_ of the half-selected cells within the bit line, and the readout margin (RM) was calculated as RM = (1 − I_sensing_/I_LRS_) × 100. The results show that the SR-SOM maintains a readout margin above 90% up to a 2^13^ × 2^13^ crossbar array, corresponding to ~67 Mbit.

**Figure S8.** Implementation of the Boolean logic gates using a single SR-SOM

| ***No.*** | ***Boolean logic*** | ***Output*** | ***Energy Consumption per Operation***  ***(Worst Case)*** |
| --- | --- | --- | --- |
| 1 | AND | $X\cdot Y$ | 4.5 pJ (X=1, Y=1) |
| 2 | NAND | $\bar{X\cdot Y}$ | 7.5 pJ (X=0, Y=1) |
| 3 | OR | X+Y | 7.5 pJ (X=0, Y=1) |
| 4 | NOR | $\bar{X+Y}$ | 4.5 pJ (X=0, Y=0) |
| 5 | XOR | $\bar{X}\cdot Y+X\cdot\bar{Y}$ | 4.51 pJ  (X=1, Y=0 & X=0, Y=1) |
| 6 | XNOR | $X\cdot Y+\bar{X+Y}$ | 7.51 pJ (X=1, Y=1) |
| 7 | IMP | $\bar{X}+Y$ | 7.5 pJ (X=0, Y=1) |
| 8 | NIMP | $X\cdot\bar{Y}$ | 4.5 pJ (X=1, Y=0) |
| 9 | RIMP | $X+\bar{Y}$ | 7.5 pJ (X=1, Y=0) |
| 10 | RNIMP | $\bar{X}\cdot Y$ | 4.5 pJ (X=0, Y=1) |
| 11 | X(transfer) | X | 4.5 pJ  (X=0, Y=1 & X=1, Y=1) |
| 12 | Y(transfer) | Y | 4.5 pJ  (X=0, Y=1 & X=1, Y=1) |
| 13 | Not X | $\bar{X}$ | 4.5 pJ  (X=0, Y=0 & X=0, Y=1) |
| 14 | Not Y | $\bar{Y}$ | 4.5 pJ  (X=0, Y=0 & X=1, Y=0) |
| 15 | True | 1 | 4.5 pJ (All cases) |
| 16 | False | 0 | 6.0 fJ (All cases) |

**Table S1.** 16 Boolean logic functions and corresponding worst-case energy consumption

| **Input logic** | | | **Output** | |
| --- | --- | --- | --- | --- |
| **X** | **Y** | **C_in_** | **Sum** | **Carry-out** |
| 0 | 0 | 0 | 0 | 0 |
| 0 | 0 | 1 | 1 | 0 |
| 0 | 1 | 0 | 1 | 0 |
| 0 | 1 | 1 | 0 | 1 |
| 1 | 0 | 0 | 1 | 0 |
| 1 | 0 | 1 | 0 | 1 |
| 1 | 1 | 0 | 0 | 1 |
| 1 | 1 | 1 | 1 | 1 |

**Table S2.** Truth table of the full adder

|  | **Components** | **Technology** | **Energy Consumption** | **Number of**  **steps / cycles** | **Ref** |
| --- | --- | --- | --- | --- | --- |
| Amini-Valashani et al. | 18 transistor | CMOS hybrid  full adder | 0.88 fJ (PDP) | 1 | [Ref. S1] |
| Shalini and Singh | 12 transistor +  6 memristor | Hybrid memristor-CMOS | 0.929 fJ (PDP) | 1 | [Ref. S2] |
| Son et al. | 10 diodes | Si p^+^-n-p-n^+^ diode | 50.4 pJ | 10 | [Ref. S3] |
| Rohani  et al. | 5 memristors | VTEAM ReRAM | 9.98 nJ | 17 | [Ref. S4] |
| Cheng  et al. | 11 memristors | Pt/HfO_2_/TiN | 0.6 nJ | 12 | [Ref. S5] |
| This work | 3 SR-SOMs | Pt/CuGeSe/GeSe/W | 15 pJ | 3 cycles (X=0) 5 cycles (X=1) |  |

**Table S3.** Benchmark comparison of 1-bit full adders

**Figure S9.** Dependency of V_th_ and RWM depending on the Cu co-sputtering power

**References**

[Ref. S1] Amini-Valashani, M.; Ayat, M.; Mirzakuchaki, S. Design and analysis of a novel low-power and energy-efficient 18T hybrid full adder. Microelectronics Journal 2018, 74, 49–59.

[Ref. S2] Shalini; Singh, K. High Performance and Scalable Hybrid Memristor-CMOS Based Full Adder. IETE Journal of Research 2024, 70 (7), 6412–6422.

[Ref. S3] Son, J.; Jeon, J.; Cho, K.; Kim, S. Stateful Full Adder Using Silicon Diodes. Advanced Intelligent Systems 2025, 7, 2400735.

[Ref. S4] Rohani, S. G.; Taherinejad, N.; Radakovits, D. A Semiparallel Full-Adder in IMPLY Logic. IEEE Transactions on Very Large Scale Integration (VLSI) Systems 2020, 28 (1), 297–301.

[Ref. S5] Cheng, L.; Li, Y.; Yin, K.-S.; Hu, S.-Y.; Su, Y.-T.; Jin, M.-M.; Wang, Z.-R.; Chang, T.-C.; Miao, X.-S. Functional Demonstration of a Memristive Arithmetic Logic Unit (MemALU) for In-Memory Computing. Advanced Functional Materials 2019, 29, 1905660.
